# Editorial: Innovative host-directed therapies for combatting antimicrobial resistance (AMR)

**DOI:** 10.3389/fcimb.2026.1949290

**Published:** 2026-08-25

**Authors:** Mariola J. Ferraro, Daniel M. Czyz

**Affiliations:** 1 Department of Microbiology and Cell Science, Institute of Food and Agricultural Sciences, University of Florida, Gainesville, FL, United States; 2 Department of Molecular Genetics and Microbiology, College of Medicine, University of Florida, Gainesville, FL, United States; 3 Emerging Pathogens Institute, University of Florida, Gainesville, FL, United States

**Keywords:** AMR (antimicrobial resistance), antibiotic, bacteria, host-bacteria inferaction, host-directed therapies (HDT), novel antibiotic, host-targeted therapeutics

Antimicrobial resistance (AMR) continues to challenge the foundation of modern infectious disease treatment. The growing prevalence of multidrug-resistant (MDR) bacterial pathogens, combined with the emergence of new resistance and stagnant development of new antibiotics, highlight the need for therapeutic strategies that extend beyond direct bacterial killing. Host-directed therapies (HDTs) offer alternative approach. Rather than targeting microbial components or pathways directly, HDTs seek to alter host responses, cellular pathways, or tissue environments in ways that improve pathogen clearance, restrict access to essential nutrients and attenuate disease severity, or restore the efficacy of existing antimicrobials. Because these therapies do not directly target rapidly evolving pathogens, they may reduce the selective pressure that drives AMR.

This Research Topic, *Innovative Host-Directed Therapies for Combatting Antimicrobial Resistance (AMR)*, was developed to identify advances in understanding how host-pathogen interactions can be exploited therapeutically. The contributions gathered under this topic emphasize a central theme: successful anti-infective therapy may require not only identifying the pathogen but also understanding the host cellular niches and immune pathways that allow pathogens to persist despite antimicrobial exposure.

A central focus of this Research Topic is the use of HDTs to combat intracellular bacterial infections, an area of growing relevance to AMR. Intracellular bacterial lifestyles can shield pathogens from antibiotics and immune defenses, creating reservoirs that contribute to recurrence, persistence, and treatment failure. van den Biggelaar et al. identified kinase inhibitors as candidate HDTs against intracellular methicillin-resistant *Staphylococcus aureus* (MRSA), a major AMR pathogen. By screening 201 ATP-competitive kinase inhibitors in a HeLa cell infection model, they identified 17 hits and selected GW633459A and GW296115X candidates for further study. Both compounds reduced intracellular MRSA across multiple human cell lines without inhibiting planktonic bacterial growth, supporting a host-directed mechanism. GW296115X activated AMPK, promoted autophagosome formation, enhanced autophagy-mediated bacterial clearance, and improved survival in MRSA-infected zebrafish embryos. These findings support host kinase signaling as a tractable HDT target for reducing intracellular bacterial burden without directly selecting for resistance, while highlighting the need to test this strategy in additional bacterial infection models, including mammalian *in vivo* systems.

The advantages and complexities of targeting host pathways to treat infection are also illustrated by Gunn et al., who investigated *S. aureus* persistence in human osteocyte-like cells during exposure to rifampicin, vancomycin, and modulators of autophagy or xenophagy. Their study addressed a clinically important problem: *S. aureus* osteomyelitis and periprosthetic joint infection can recur despite antibiotic therapy, in part because bacteria adapt to intracellular bone cell niches and slow-growth states. Using a human osteocyte-like infection model, the study examined if antibiotic-mediated disruption of autophagy contributed to persistent intracellular infection. Rifampicin, alone or with vancomycin, rapidly reduced intracellular bacterial culturability; however, bacterial DNA levels remained stable or increased, suggesting that antibiotic treatment diminished colony-forming capacity without fully eliminating the intracellular bacterial burden. Both antibiotics also inhibited autophagic flux, revealing unintended host-directed effects of conventional therapy. Yet experimental modulation of autophagy did not fully explain the persistence phenotype. This study shows that host-directed therapy requires more than selecting a host pathway to manipulate; effective intervention must consider the infected cell type, pathogen physiology, antimicrobial exposure, and timing of treatment.

Li et al. extend this osteomyelitis theme by identifying the host Mprip/Talin-1 interface as a mechanism that promotes intracellular *S. aureus* persistence. Using bone marrow mesenchymal stem cells and a murine osteomyelitis model, they showed that recruitment of Mprip to Talin-1 drives Talin-1 phosphorylation, bacterial internalization, and impaired osteogenic recovery through PI3K/AKT signaling. Genetic disruption of Mprip or Talin-1 reduced bacterial burden and restored osteogenesis. The authors also identified UM-164, a small molecule that disrupts the Mprip/Talin-1 interaction, limits bacterial internalization, and synergizes with gentamicin to reduce bacterial burden and bone destruction *in vivo*. This study shows how targeting host machinery exploited by pathogens can enhance conventional antibiotic efficacy.

Finally, Adani et al. developed a cell-based high-throughput screening platform using an intracellular-induced lux reporter to identify compounds that inhibit host cell infection by intracellular pathogens. Their screen of nearly 37,000 bioactive small molecules yielded eight non-cytotoxic compounds that inhibited intracellular *Salmonella enterica* serovar Typhimurium growth in human epithelial cells without affecting bacterial growth in culture, suggesting activity at the host-pathogen interface rather than direct antibacterial activity. Several hits were also active in primary mouse macrophages, indicating that the effects were not limited to a single cell line. Remarkably, seven of the eight compounds also inhibited intracellular growth of the Gram-positive pathogen *Listeria monocytogenes*. This cross-pathogen activity suggests that some HDT candidates may target conserved host processes used by phylogenetically distinct intracellular pathogens. The study also emphasizes the value of infection-relevant screening platforms that preserve host cell biology, bacterial intracellular behavior, and compound safety considerations during early discovery.

Translation of these largely cell-based observations into therapies usable in patients will require a staged validation pathway. Although cell culture systems are indispensable for identifying HDT candidates and defining mechanisms, they cannot reproduce the tissue architecture, multicellular immune responses, pharmacokinetics, drug penetration, or on-target host toxicity that shape treatment outcomes *in vivo*. The zebrafish embryo data reported by van den Biggelaar et al. and the murine osteomyelitis model used by Li et al. provide important proof-of-concept bridges. Yet, these and other mechanistic findings require testing in animal models more extensively. Future studies should assess efficacy in mammalian models that recapitulate persistent and tissue-specific infection, determine whether HDTs improve antibiotic treatment at clinically achievable exposures, and define effects on host physiology, immune function, and tissue repair.

Together, these studies demonstrate both the promise and complexity of HDT strategies to mitigate AMR. They point to several important principles for the field. First, intracellular infection models are essential for identifying therapies that conventional broth-based antimicrobial screens may miss. Second, host pathways such as kinase signaling, autophagy, xenophagy, adhesion signaling, and cytoskeletal regulation can be manipulated to alter infection outcomes, but their effects may vary depending on the pathogen, host cell type, infection niche, and timing of intervention. Third, distinguishing host-directed effects from direct antimicrobial activity remains essential, especially when considering drug repurposing or small-molecule screening approaches. Finally, translational progress will require attention to safety, cell specificity, dosing, and the potential consequences of altering host pathways that also support normal host physiology.

At this point, HDTs are unlikely to replace antibiotics in the near term. Instead, their value may lie in complementing existing antimicrobial strategies, improving clearance of persistent intracellular infections, and reducing the selective pressure that drives resistance. By focusing specifically on host-pathogen interactions, this Research Topic contributes to a much-needed and emerging field of anti-infective strategies in which the host is not merely the site of infection, but an active therapeutic target and participant. Continued progress will depend on interdisciplinary approaches that integrate microbiology, immunology, cell biology, and clinically relevant infection models. The studies collected here show how such approaches can generate new therapeutic hypotheses and candidate interventions for difficult-to-treat bacterial infections.

